# Clustering-Based Thermography for Detecting Multiple Substances Under Large-Scale Floating Covers

**DOI:** 10.3390/s24248030

**Published:** 2024-12-16

**Authors:** Yue Ma, Benjamin Steven Vien, Thomas Kuen, Wing Kong Chiu

**Affiliations:** 1 Department of Mechanical & Aerospace Engineering, Monash University, Clayton, VIC 3800, Australia; ben.vien@monash.edu (B.S.V.); wing.kong.chiu@monash.edu (W.K.C.); 2 Department of Integrated Planning, Melbourne Water Corporation, 990 La Trobe Street, Docklands, Melbourne, VIC 3008, Australia; thomas.kuen@melbournewater.com.au

**Keywords:** thermal imaging monitoring, structural health monitoring, image segmentation, high-density polyethylene geomembranes, floating covers, water treatment plant

## Abstract

This study presents a novel approach for monitoring waste substrate digestion under high-density polyethylene (HDPE) geomembranes in sewage treatment plants. The method integrates infrared thermal imaging with a clustering algorithm to predict the distribution of various substrates beneath Traditional outdoor large-scale opaque geomembranes, using solar radiation as an excitation source. The technique leverages ambient weather conditions to assess the thermal responses of HDPE covers. Cooling constants are used to reconstruct thermal images, and clustering algorithms are explored to segment and identify different material states beneath the covers. Laboratory experiments have validated the algorithm’s effectiveness in accurately classifying varied regions by analyzing transient temperature variations caused by natural excitations. This method provides critical insights into scum characteristics and biogas collection processes, thereby enhancing decision-making in sewage treatment management. The methodology under development is anticipated to undergo rigorous evaluation across various floating covers at a large-scale sewage treatment facility in Melbourne. Subsequent to field validation, the implementation of an on-site, continuous thermography monitoring system is envisioned to be further advanced.

## 1. Introduction

**Figure 1:**
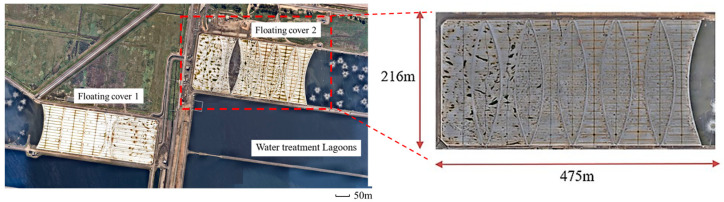
Satellite view of floating HDPE geomembrane covers in covered anaerobic lagoons (CAL) at the wastewater treatment plant in Melbourne Water, Australia. Both lagoons are covered by 2 mm thick HDPE geomembrane. The size of both covers is 216 m × 475 m.

**Figure 2:**
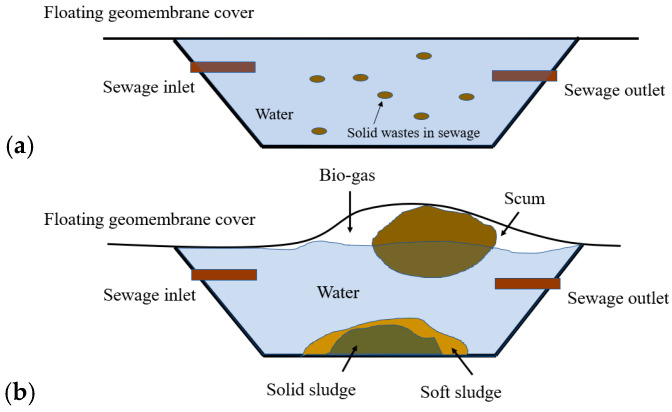
Illustration of the digestion process at the CAL. (**a**) Condition of the lagoon when sewage water is pumped from the inlet. (**b**) Condition of the lagoon after the sewage water digestion.

High-density polyethylene (HDPE) geomembranes are widely deployed at sewage water treatment plants with high corrosion resistance, durability, and impact strength [1,2]. As shown in Figure 1, multiple anaerobic lagoons are located in sewage treatment plants operated by Melbourne Water Corporation (MWC), Australia, to treat sewage from the city. The first lagoon from the inlets is covered by large sheets of HDPE geomembranes. The covered anaerobic lagoons (CAL) provide an anaerobic environment for bacteria to digest the raw sewage into biogas and sludge. These HDPE geomembranes, also known as floating covers, rest on the surface of the sewage water with each cover spanning an area of approximately 216 m × 475 m. These floating covers play an important role in collecting generated biogas as renewable energy and preventing the emission of odorous greenhouse gases. Unfiltered sewage water is first pumped into the lagoons for preliminary sedimentation of the solids. During the anaerobic digestion, the flow of the sewage water slows down after being pumped into the from 6- to 8-m-deep lagoon, where solid elements in the raw sewage water are broken down into biogas and sludge by bacteria as illustrated in Figure 2a. Some undigested light sludges and floatable solids combine into a large body of “scum”, which floats on the water’s surface. Over time, the volume of scum can accumulate, lifting the HDPE floating cover and affecting the collection of biogas. As shown in Figure 2b, scum can accumulate into “scumbergs”, which float like icebergs on the lagoon, deforming the shape of the floating cover. Accumulated scum can elevate the surface of the floating cover up to 1–2 m, leading to lateral strain in the scum region [3,4]. In addition, when the scum moves close to the inlet or outlet of the lagoon, it can block the biogas pathway and reduce the efficiency of sewage water digestion [5,6]. Currently, MWC conducts a series of activities to ensure the structural integrity of its assets. For example, an unmanned aerial vehicle (UAV) has been deployed at the plant to quantify the covers’ deformation state [7,8]. Wong et al. utilized a UAV to scan the elevations of floating covers at MWC and generated digital elevation models (DEMs) through the UAV scans, thus allowing for the estimation of the distribution of subsurface scum and identification of wrinkles on the covers by comparing the DEM at different times in a year [8]. In addition to UAV–assisted surveys, Vien et al. employed a finite element analysis by importing the DEM map, estimating the distribution of strains and stress on the covers based on the obtained geomembrane altitude information [3,9]. While the correlation between the geomembrane cover elevation and scum depth can be identified using UAV surveys and image segmentation methods with elevation data, distinguishing between different substrate states beneath the cover remains challenging [10]. Apart from previous investigations, continuing research is to be carried out to better distinguish multiple states of objects beneath the covers, enabling a more rigorous assessment.

Thermal imaging is a widely accepted technique for structural health monitoring. The process of converting infrared radiation into visible images depicts the spatial distribution of temperature differences in a scene viewed by a thermal camera. This technique has been used in non-destructive monitoring studies for concrete bridges, aerospace structures [11], and composite structures [12]. Thermal imaging can be categorized into active and passive methods, depending on the inspection approach. Active thermal imaging [13] requires an artificial heat source to introduce thermal waves into the deeper part of substances, with the defective regions appearing in the thermal image due to the blockage of thermal energy [14]. The thermography signal reconstruction (TSR) method is particularly effective in enhancing monitoring quality. In active thermography, thermal images are enhanced by increasing contrast through curve fitting the temperature curve of the pixels [15], and anomaly regions are identified by comparing the cooling rate of temperatures. The TSR method also identifies anomalies using the Logarithmic Peak Second-order Derivative Method (LPSD) or through locating maximum data points [16,17,18]. However, it is less effective at distinguishing substances with similar temperature-changing rates. Given that active thermography utilizes external heat sources as excitations, it faces limitations in providing uniform area excitation, resulting in inefficient large-area structural assessment and insufficient temperature response contrast among different regions. In contrast, passive thermal imaging [19] relies on the self-heating of the monitored structures. Since it does not need an external heat source, this technique is suitable for large-scale structures, such as bridges, pavements, and pipelines, where the defective region can be detected based on temperature differences from their surroundings. Omar and Nehdi [20] used a UAV–mounted thermal camera to scan a concrete bridge, rapidly assessing its condition by mapping the temperature distribution. In addition, they mounted the thermal camera on a moving platform to scan another bridge, stitching the temperature maps together to produce a comprehensive condition map that delineates different kinds of delamination [21]. However, current passive thermography methods still struggle to identify substrate materials with similar material properties.

Outdoor thermography on civil structures is currently limited by ambient environmental factors such as atmospheric temperature, solar intensity, and emissivity variation due to time of monitoring, which can result in low-temperature contrast among different regions. In addition, image segmentation results based on randomly selected thermal image frames from passive thermography can vary depending on the temperature value contrasts. At certain times, two objects may have the same temperature and thus be classified as the same object. Moreover, the subjective selection of the threshold for the cooling constant map during thermal image reconstruction or processing can limit the identification of regions on the cover. Integrating thermography with segmentation methods can aid in identifying substances. Therefore, to enhance monitoring results, outdoor thermal images should be reconstructed from a series of thermal image sequences rather than single frames, with segmentation based on the temperature change rate rather than absolute temperature values.

Machine learning-based image segmentation algorithms present an opportunity to enhance thermal imaging results by grouping data into meaningful clusters based on intrinsic similarities. Several supervised deep learning segmentation models, such as U-Net, Res-Net [22,23], and YOLO (You Only Look Once) [24,25] have been used for structural health monitoring. In particular, U-Net architecture has been widely deployed with thermography to identify different features and structural defects with its symmetric architecture and expansive paths [15,16,26,27]. However, these supervised models require large sets of training data [17]. Labeling or predefining the regions of the covered substrates is challenging, as the contact areas cannot be measured before the detection and change immediately after the cover is lifted. Alternatively, unsupervised clustering-based algorithms can be used for segmentation. This algorithm requires the data to find its structure, and the results are unknown beforehand. Clustering-based algorithms, such as the K-means algorithm, the Density-based spatial (DBSCAN) algorithm, and the Gaussian mixture model (GMM) algorithm have been used to segment thermal images by segmenting the data into clusters that represent different temperature ranges [18,28,29]. These segmentation methods not only amplify the distinction between regions of interest but also help in noise reduction by isolating irrelevant variations. Furthermore, clustering techniques enable the customization of thermal imaging according to specific applications, as they can learn from data to recognize patterns related to faults, leaks, or other anomalies. Thus, by providing a data-driven, adaptive approach to image analysis, clustering-based machine learning algorithms offer a sophisticated tool for enhancing the interpretability of thermal imaging. Here, the image segmentation technique is performed as a thresholding classification tool to arrange the data into categories. Data clustering has been widely used in the field of RGB image segmentation [30,31].

K-means clustering [32] is an unsupervised distance-based partitioned clustering method that aims to divide a dataset into *K* clusters. It has been widely used in statistical analysis and is very efficient. K-means clustering aims to characterize the similarity of data and organize the data into a predefined number of *K* clusters. The algorithm converges when the centroids no longer change significantly or when the change in the within-cluster sum of squares (WCSS) is below a certain threshold. The WCSS is given by:

(1) $$WCSS={\sum_{j=1}^{K}\sum_{x_{i\in }S_{j}}\left||x_{i}-c_{j}|\right|}^{2}$$

where *K* is the number of clusters, $x_{i}$ represents a data point in the dataset, $S_{j}$ is the set of all points assigned to the cluster j, and $c_{j}$ is the centroid of cluster j.

Here, the algorithm defines an initial mean vector for each group, and data are assigned to a group to minimize the squared error between the point data and the empirical mean of the group. Then, a new set of vectors is updated, and the iteration continues until the assignment of the points converges. K-means clustering is functional when the number of clusters k is known, but the noise pixels in the image would affect the result of clustering [33,34]. Lu et al. [35] detected the micro solder balls using active thermography and the K-means algorithm. To enhance the signal-to-noise ratio, a combination of polynomial fit and differential absolute contrast techniques was deployed to reconstruct the thermal images. Further, the statistical attributes corresponding to each solder ball were extracted from these reconstructed images and employed for clustering analysis utilizing the K-means algorithm. The empirical results from this study affirm that all solder balls were accurately identified. Yin et al. [36] captured thermal images of a photovoltaic (PV) module and subsequently analyzed them via a MATLAB–implemented Hotspot Detection algorithm. The K-means algorithm segmented the processed thermal image map; it proffered both average temperature and relative area to quantify the hotspot. This study also encompassed various features and conditions precipitating hotspots, including cracks, junction box anomalies, and shading effects. Omar and Nehdi [37] conducted in situ infrared testing on a full-scale bridge deck. Using image analysis predicated on the K-means clustering technique, the bridge deck was segmented to identify different defect groups, generating a condition map that categorizes varying degrees of delamination severity. This map was further corroborated through ancillary techniques applied to the same bridge. DBSCAN [38] is one kind of Density-based clustering method. It clusters the data based on a threshold for a neighborhood search radius (ε) and a minimum number of neighborhood points (minpts) required to find out a core point. The cluster will be created after the core points are defined and the neighborhood points will be checked in the created clusters [39]. The process will be repeated until no points can be added to clusters. The DBSCAN algorithm is good at processing nonlinear data, and it does not need to identify the number of clusters before the procession [40,41]. But it struggles to process large-density data, and the number of clusters cannot be controlled. Ngo et al. utilized the DBSCAN algorithm to isolate the hotspot of PV systems in thermal imaging [42]. In Gaussian mixture model (GMM) clustering, the dataset can be regarded as a multivariate distribution that consists of multivariate Gaussian distribution components, and each of the components is determined by the covariance and the mean [43]. The GMM model has been used as a probabilistic method in image processing. The key advantage of GMM in image segmentation is its flexibility in modeling a wide range of pixel intensity distributions, making it suitable for complex images [44,45]. Similar to K-means clustering, the number of components in the GMM model needs to be determined before the classification.

The objective of this paper is to devise a clustering-based thermographic approach aimed at segmenting disparate subsurface features beneath HDPE geomembranes. The investigation is intended to facilitate the predictive mapping of the spatial distribution of these subterranean characteristics, thereby enhancing the understanding and assessment of the geomembrane’s integrity. In this paper, unsupervised clustering algorithms are used to perform image segmentation on the reconstructed thermal image to define different regions on a geomembrane surface in contact with multiple substances such as scum, biogas, and sewage water beneath. The methodology commences with the utilization of ambient solar radiation excitations that cause transient temperature responses within the cover, followed by the capture of thermal images in synchronization with geomembrane temperature fluctuations. These images are then subjected to rigorous preprocessing to enhance monitoring quality, and temperature curves are constructed from the sequential thermal images. Newton’s cooling law [46] is used to derive the cooling constant for each pixel, and these values are assembled and clustered using algorithms such as K-means, DBSCAN, and GMM. The clustered data are reallocated to corresponding pixels and remapped, segmenting regions on the geomembrane covers delineated by cooling constant values. This intricate process facilitates the prediction of the distribution of substances beneath the cover, providing a nuanced understanding of the underlying substances, and holds significant potential for applications in environmental monitoring and waste management.

## 2. Thermal Imaging Method

### 2.1. Heat Transfer Model

**Figure 3:**
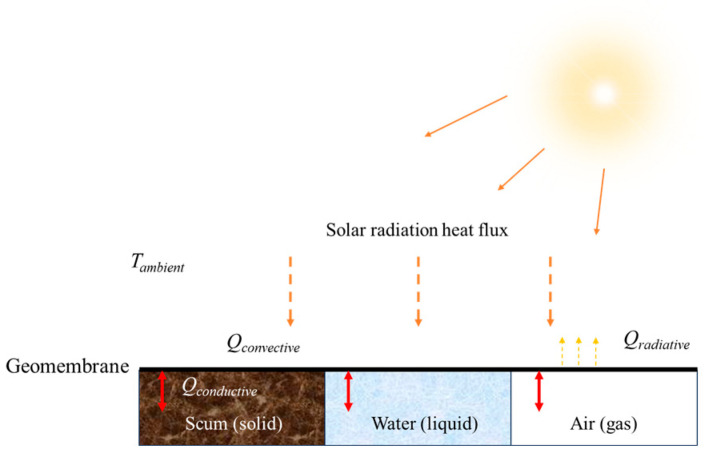
Illustration of heat transfer process between the geomembrane and ambient.

This paper investigates a method of using thermal imaging to find out the different states of substances under HDPE geomembrane floating covers. The proposed method uses ambient weather information, which includes ambient temperature and solar intensity to compare the temperature-changing rate of the cover and find out regions with gas, fluid, and solids under the cover. To identify the existence of different substances under the opaque cover, the heat transfer balance needs to be discussed first. When air (gas) or water (liquids) contact with the subsurface of the geomembrane, thermal convection happens between the interfaces, while thermal conduction happens between the interfaces when the geomembrane contacts with the soil (solids). In a cross-sectional view, the relevant boundary conditions for the model are indicated in more detail in Figure 3.

The convection and radiation heat flux from the top surface of the geomembrane can be expressed by:

(2) $$Q=Q_{convective}+Q_{radiative}$$

where Q is heat energy exchanged between the surface of the geomembrane, the environment $Q_{convective}$ is the heat energy exchanged by convection, and $Q_{radiative}$ is the heat energy exchanged by radiation. The convective heat transfer at the upper surface of the geomembrane $Q_{convective}$ is revealed by Newton’s law:

(3) $$Q_{convective}=h_{c}\left[T(t)-T_{ambient}(t)\right]$$

where T(t) and $T_{ambient}$ denote the membrane temperature and ambient temperature at a time instant t, and $h_{c}$ is the convective heat transfer coefficient. Radiative heat transfer for the geomembrane satisfies the Stefan–Boltzmann law as follows:

(4) $$Q_{radiative}=\varepsilon \sigma (T^{4}-T_{ambient}^{4})$$

where T and $T_{ambient}$ are interpreted as absolute temperatures, σ is the Stefan–Boltzmann constant, and ε is the emissivity, which is equal to the absorptivity α according to Kirchoff’s law. In general, Equation (4) requires the use of a reduced emissivity, which combines the emissivity of the cover and the ambient emissivity. However, in this specific case, both emissivity values are nearly identical and close to unity. As a result, the difference between the two is negligible, justifying the use of a single emissivity value in Equation (4) without significant loss of accuracy. For the range of temperatures encountered in this context, the difference T − $T_{ambient}$ is relatively small compared with the absolute ambient temperature $T_{ambient}$. Therefore, Equation (3) can be rewritten in the form:

(5) $$Q_{radiative}=h_{r}\left[T(t)-T_{ambient}(t)\right]$$

where $h_{r}$ is radiative heat transfer coefficient and can be expressed as:

(6) $$h_{r}\approx 4\varepsilon \sigma T_{ambient}^{3}$$

Accordingly, Equation (1) can now be expressed as:

(7) $$Q=h\left[T(t)-T_{ambient}(t)\right]$$

where h is the heat transfer coefficient and can be expressed as:

(8) $$h=h_{c}+h_{r}$$

To simplify the model, it is assumed that there is no heat transfer through the external boundaries on the bottom side of the membrane, represented as Q = 0, as depicted in Figure 3. However, the membrane is considered to have ideal thermal contact with the soil substrate within the soil area, meaning that both temperature and heat transfer are continuous across this boundary.

Following this, the next stage in determining the cooling kinetics involves estimating the Biot number:

(9) $$Bi=\frac{hL_{c}}{k}$$

Here, k represents the thermal conductivity, and $L_{c}$ denotes an appropriate characteristic length, which in this instance can be taken as the thickness of the membrane. This yields a value of Bi ≈ 0.06. Since this value is lower than 0.1, it is safe to disregard the temperature gradient across the membrane thickness. Consequently, the heat balance equation for a geomembrane on air (or biogas) can be expressed in the following manner:

(10) $$\rho CL\frac{dT(t)}{dt}=h\left[T(t)-T_{ambient}(t)\right]$$

where ρ, C, and L refer to the density, specific heat, and thickness of the membrane, respectively. It is assumed that the ambient temperature is constant or varies slowly over time, and (7) can be integrated as:

(11) $$T(t)=T_{ambient}+(T_{i}-T_{ambient})e^{-bt}$$

where

(12) $$b=\frac{C}{hA}$$

In this context, the symbol $T_{i}$ represents the initial temperature and *A* is the heat transfer area, while *b* denotes the cooling constant. The cooling constant is presented without a dimensional unit because it is treated as a dimensionless parameter in the context of the model. The cooling constant is derived from a time-dependent temperature decay model and represents the relative rate of temperature change. It is a characteristic of the system that depends on material properties and environmental factors, rather than being associated with a specific dimensional unit. As such, it is typically expressed in a normalized form, where the time scale is non-dimensionalized relative to system-specific parameters. Therefore, the absence of a dimensional unit for the cooling constant reflects its usage in this dimensionless context. It should be noted that (8), which exhibits an exponential decay pattern, is strictly applicable only to the geomembrane in contact with air or biogas. Nevertheless, it is feasible to determine a value for the cooling constant in regions where the geomembrane is in contact with soil, which simulates scum. This determination can be achieved by fitting the thermal transients, measured or calculated, to an exponential decay law.

After a derivation, (11) becomes:

(13) $$\frac{dT(t)}{dt}=b(T(t)-T_{ambient}(t))$$

From this equation, it can be found that if the temperature of the observed substance and the environment temperature are provided, it can be used to estimate the temperature-changing rate of substances in transient temperature change events. Given that the temperatures of HDPE geomembrane floating covers and ambient temperature vary according to the power of solar radiation in a day, Newton’s cooling law is used to identify scum, biogas, and sewage water under the cover by comparing them in a day. This method is more accurate than simply comparing temperature as a guide to distinguish substances under the cover because it analyses the temperature response of the geomembrane based on long-term transient temperature changes and does not focus on a single frame of the randomly selected thermal image.

### 2.2. Signal Processing Methodology

**Figure 4:**
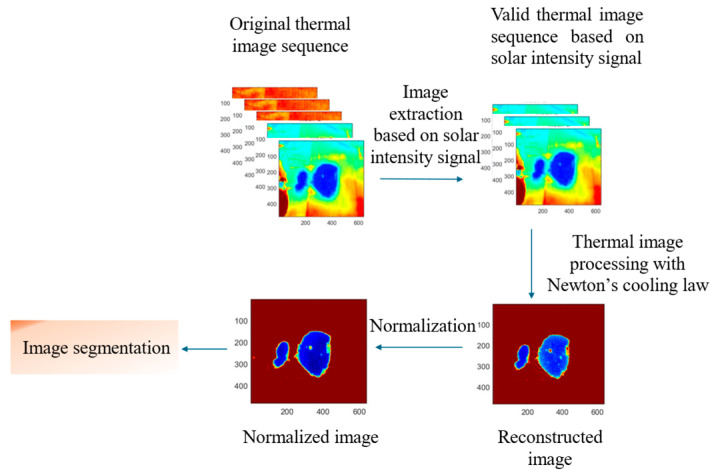
Flow chart of image procession.

Traditional outdoor thermography, which relies on ambient solar radiation as an excitation source, may face limitations in climates with varying solar radiation levels, potentially impacting the accuracy of temperature-based clustering. To mitigate the influence of solar radiation in our methodology, both the solar radiation signals and the corresponding temperature variations of the geomembrane were recorded. By applying Newton’s cooling law, the temperature profiles throughout the solar radiation changes are analyzed, utilizing a single benchmark cooling constant to represent the entire temperature variation process. The utilization of the cooling constant, derived from Newton’s cooling law, has been incorporated into the thermographic approach to encapsulate the testability under varying environmental conditions. This strategic application of the cooling constant serves to mitigate the influence of transient temperature distributions, which are subject to the stochastic fluctuations of solar radiation and ambient temperature. By employing the cooling constant, this method transcends the constraints imposed by temperature readings at arbitrary intervals, offering a holistic and representative assessment of the thermal dynamics beneath HDPE geomembranes. The cooling constant, as a dimensionless parameter, normalizes the temperature decay rate, providing a robust measure that is less sensitive to instantaneous temperature values and more reflective of the underlying thermal properties of the substances beneath the geomembranes. The proposed approach can eliminate inaccuracies associated with segmentation results derived from single-frame thermal images, thereby enhancing the reliability of segmentation outcomes. A single frame of the unprocessed thermal image has difficulties defining the anomaly region or defining different interfaces under the geomembrane. As the temperature of the geomembrane changes according to the environment during the day, the temperature contrast on the surface of the geomembrane will become large during warmer periods and small during cool periods [17]. A contour plot of the temperature distribution is insufficient to classify different regions and calculate the corresponding areas. Therefore, an image process procedure is applied as shown in Figure 4. Recorded thermal images are first imported into MATLAB R2023a (MathWorks, Natick, MA, USA) and the collected temperature information in the thermal image will be reconstructed as a map of the cooling constant with the algorithm based on Newton’s cooling law.

After normalization, the clustering algorithms are used to segment the obtained cooling constant maps from experiments.

Segmentation parameters, such as the number of clusters K in the K-means algorithm, a threshold for a neighborhood search radius and a minimum number of neighborhood points in the DBSCAN algorithm, and the number of components in the GMM algorithm, need to be determined according to the segmentation requirement. In this study, it is required to segment solid, liquid, and gas regions on the surface of the covers. Therefore, the number of clusters was set as 3. Cluster numbers were also set as 4 and 5 to compare the segmentation results. The cooling constant values of each pixel stored in the cooling constant map are transferred into a vector. After the segmentation, the classified values are re-input into the cooling constant matrix to reconstruct a new map that can indicate the distribution of each cluster, and the resultant processed images are compared to evaluate the classification quality of each method.

## 3. Thermal Imaging Monitoring on the HDPE Geomembrane

### 3.1. Setting up Weather Recording–Thermal Monitoring System

**Figure 5:**
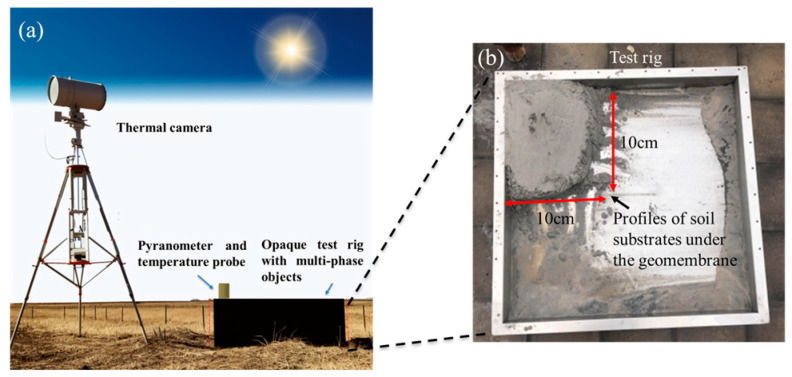
Illustration of the experiment set-up. (**a**) Outdoor thermography set up. (**b**) Profiles of soil block in the test rig.

The HDPE geomembrane has been proven to have high emissivity and it is suitable for thermal imaging tests [17]. A preliminary lab-scale experiment was set up outside. As shown in Figure 5a, a thermal camera was fixed on a frame to constantly monitor a sheet of the HDPE geomembrane, the same material as the floating covers at anaerobic lagoons. An opaque aluminum test rig was deployed and filled with different substances. The HDPE geomembrane was used to cover the top of the rig to act as the floating covers. As shown in Figure 5b, in the preliminary experiment, a 10 cm × 10 cm soil block was placed in the test rig, and the soil block’s body was in contact with the subsurface of the HDPE geomembrane. As the clayey soils are known to have similar thermal properties, such as density [47], thermal conductivity [48,49], and specific heat [50,51], as the scum which is difficult to extract from under the floating covers, the clayey soil was used to replace the scum as the solid substance under the geomembrane.

**Figure 6:**
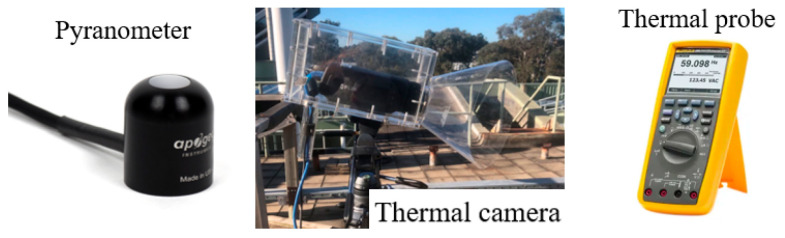
Experiment with equipment of outdoor thermal imaging monitoring system.

Figure 6 shows the equipment that was used in the experiment. A high-resolution IR thermal camera, FLIR A615 (Teledyne FLIR, Wilsonville, OR, USA) [52], was deployed to monitor the temperature evolution of the geomembrane [17]. The sampling rate of the thermal camera was set as 10 s/frame, in this paper. An Apogee SP-110 pyranometer (Apogee Instruments, Logan, UT, USA) [53] was used to record the local solar intensity. Simultaneously, a thermal probe from a Fluke 287 multi-meter (Fluke, Everett, WA, USA) [54] was used to record the ambient air temperature. The manufacturer data of the instruments are presented in Table 1. Considering the experiments were executed in an outdoor environment, the ambient temperature fluctuated between 10 °C and 25 °C, the temperature of the geomembrane ranged from 0 °C to 50 °C, and the solar radiation intensity varied from 0 to 1000 W/m^2^. Under these conditions, the acquired signal is deemed suitable for the intended measurements. In this experiment, the pyranometer and the thermal probe played together as a real-time weather monitoring system to record the solar intensity signal and air temperature signal, where the thermal camera recorded the temperature response of the geomembrane under the change of weather. The set-up was exposed outside for two days to find out the desired monitoring period during the day. All instruments were set to start recording simultaneously to match the time of measured data, and cooling constant maps were reconstructed for different periods in a day.

### 3.2. Investigation of the Suitable Thermal Imaging Monitoring Period with Ambient Weather Information

**Figure 7:**
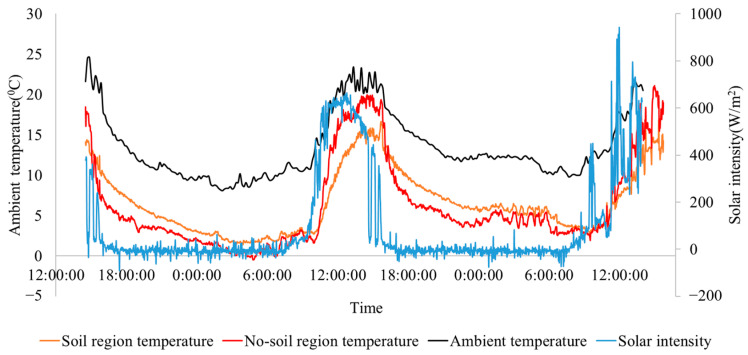
Recorded experiment data (solar intensity, ambient temperature, and temperature profiles of a randomly selected point on the soil region and a randomly selected point on the non-soil region on the geomembrane).

To validate the reliability of the technique, the experiment was carried out in the winter when the solar intensity and ambient temperature were low, with which the daily temperature variation of the geomembrane was small. As mentioned above, a desirable monitoring period or thermal image sequence needs to be identified from the experiment data. As shown in Figure 7, given that the position and the area of the subsurface soil block were known, the temperature profile of a randomly selected point in the soil region and a randomly selected point in the non-soil region was demonstrated. The local solar intensity is also plotted to verify that geomembrane temperature changes with the ambient environment condition. The acquired thermal image sequences were collected from the thermal camera, and the temperatures of the monitored region were calculated using (4). Given that the sampling rate of the thermal camera was set as 10 s/frame, cooling constants were calculated every 10 s. Then, 10 sample points were randomly selected on the membrane (5 on the soil region and 5 on the non-soil region), and the averaged absolute values of cooling constants for these points over the whole experiment were calculated.

**Figure 8:**
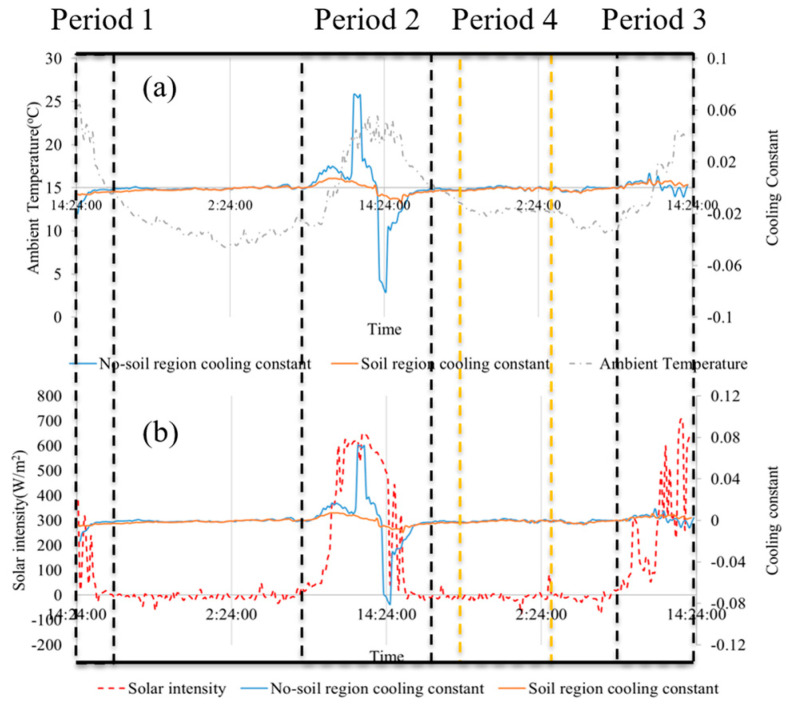
Illustration of cooling constants on soil region and non-soil region with the change of solar intensity and ambient temperature. (**a**) Local ambient temperature history and cooling constant profiles. (**b**) Local solar intensity history and cooling constant profiles.

To correlate the cooling constant of the geomembrane with the weather information, the profile of ambient air temperature profile was plotted, as shown in Figure 8a, and the solar intensity history was plotted, as shown in Figure 8b. These two figures reveal that cooling constants of regions on the geomembrane were large during the daytime (13:01:13–14:11:15 in day 1, 07:07:55–17:11:15 in day 2, and 07:41:15–14:01:15 in day 3), where the solar intensity was not equal to 0 while, after sunset, where the solar intensity was equal to 0, the cooling constants of points in all regions were approximately equal to 0. This is because of the lack of heat stimulus during the night. Although the geomembrane is still cooling during the night (see Figure 7), the tiny difference in cooling constants between the soil and non-soil regions is not helpful. It is noted that ambient temperature will change according to the solar intensity as the environment receives the heat from the sunlight, and the ambient air temperature will be used to calculate the cooling constant in (11).

**Figure 9:**
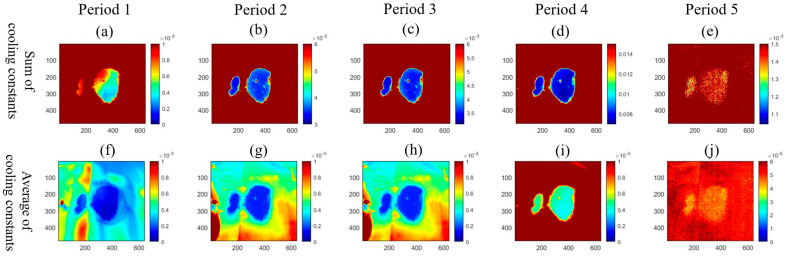
Reconstructed cooling constant maps at different periods in the two-day experiment. (**a**–**e**) The sum of cooling constants from thermal image sequences. (**f**–**j**) Average of cooling constants from thermal image sequences. (**a**,**f**) Period 1. (**b**,**g**) Period 2. (**c**,**h**) Period 3. (**d**,**i**) Periods 1, 2, and 3. (**e**,**j**) Period 4.

To validate the reliability of the technique, the experiment was carried out in the winter when the solar intensity and ambient temperature were low, with which the daily temperature variation of the geomembrane was small. As mentioned above, a desirable monitoring period or thermal image sequence needs to be identified from the experiment data. As shown in Figure 8, given that the position and the area of the subsurface soil block were known, the temperature profile of a randomly selected point in the soil region and a randomly selected point in the non-soil region was obtained. The local solar intensity is also plotted, to verify that geomembrane temperature changes with the ambient environment condition. The acquired thermal image sequences were collected from the thermal camera, and the temperatures of the monitored region were calculated using (2). Given that the sampling rate of the thermal camera was set as 10 s/frame, cooling constants were calculated every 10 s. Then, 10 sample points were randomly selected on the membrane (5 on the soil region and 5 on the non-soil region), and the averaged absolute values of cooling constants for these points over the whole experiment were calculated. To correlate the cooling constant of the geomembrane with the weather information, the profile of ambient air temperature profile was plotted, as shown in Figure 9a, and the solar intensity history was plotted, as shown in Figure 9b. These two figures reveal those cooling constants of regions on the geomembrane were large during the daytime (13:01:13–14:11:15 in day 1, 07:07:55–17:11:15 in day 2, and 07:41:15–14:01:15 in day 3), where the solar intensity was not equal to 0 while, after sunset, where the solar intensity was equal to 0, the cooling constants of points in all regions were approximately equal to 0. This is because of the lack of heat stimulus during the night. Although the geomembrane is still cooling during the night (see Figure 8), the tiny difference in cooling constants between the soil and non-soil regions is not helpful. It is noted that ambient temperature will change according to the solar intensity as the environment receives the heat from the sunlight, and the ambient air temperature will be used to calculate the cooling constant in (2).

Four regions were highlighted from Figure 8, and the suitable monitoring periods were investigated with maps of cooling constant. Several findings of this experiment are summarized below:

- Cooling constants on both the soil region and non-soil region were large during the daytime, and they increased significantly in the morning with the increase in solar intensity. While cooling constants decreased at noon, the solar intensity remained stable at this time. After the minimum point, cooling constants increased again with the increased solar intensity changing rate.
- The contrast of cooling constants between the soil and non-soil regions was around 0 when the solar intensity remained stable, and the contrast became significant when the solar intensity changed abruptly. Therefore, it is reliable to conduct thermal imaging monitoring during the high solar intensity changing rate period, and the contrast of cooling constants at different regions can help distinguish the interfaces at the underside of the geomembrane.
- The cooling constant of the soil region remained stable in the daytime, which was the result of the thermal conduction from the underside of the geomembrane to the soil, where the heat from the sunlight was stored in the soil. While the cooling constant on the non-soil region changed fast with the variation of solar intensity, the heat was stored in the geomembrane since the thermal convection efficiency between the underside of the geomembrane and the air was low.

To identify the soil profile under the geomembrane, cooling constants were calculated from the thermal image sequences with (2), and values of cooling constants were summed up to enlarge the contrast and uncover the soil regions. As shown in Figure 9, several cooling constant maps were reconstructed from the four highlighted periods in Figure 8. As shown in Figure 9a–e, the cooling constants of each pixel for every 10 s were accumulated over time, and the maps were reconstructed from over different periods. The averaged cooling constants over these periods are used to reconstruct the maps, as shown in Figure 9b–f. Figure 9a,f was reconstructed from period 1 (13:01:13–14:11:15) in Figure 8; Figure 9b,g was reconstructed from period 2 (07:07:55–17:11:15) in Figure 8; Figure 9c,h was reconstructed from period 3 (07:41:15–14:01:15) in Figure 8; Figure 9d,i was reconstructed from period 1, 2, and 3 in Figure 8, and Figure 9e,j was reconstructed from period 4 (17:21:15–01:21:15) in Figure 8. Since cooling constant maps were reconstructed with different lengths of thermal image sequences, the scales of the maps were set individually for each map. In Figure 9a, it can be observed that an overall profile of soil was presented in the cooling constant map for period 1, but a small region of soil is blurred in the figure. In addition, the soil region’s features are unclear and the contrast between the soil region and the non-soil region is small. In Figure 9b,c, from periods 2 and 3, the contrasts between regions become significant and the features of the soil regions are clearer. This is because these figures were calculated from a longer thermal image sequence, and the cooling constant differences accumulate over time. In Figure 9d, the profile of the soil region is most obvious with the thermal images from periods 1, 2, and 3 calculated. However, in Figure 9i, it is difficult to recognize the soil regions from the background due to the small difference between the soil region and non-soil region in low solar intensity changing rate period. The summation of the cooling constant can result in a blurred map, which is not helpful to identify the interface under the geomembrane. Regarding the average cooling constant map in Figure 9f–j, similar to the maps of summation of cooling constants, longer thermal image sequences will help identify the profiles of soil regions, and the result of period 4 also does not help. While the overall profiles of soil regions in averaged cooling constant maps are not as clear as in cooling constants summation maps, this is because the contrast of averaged cooling constant map does not accumulate over time, and the sum of the cooling constant values contrast is larger than the averaged values contrast. Hence, it can be concluded from this experiment that thermal imaging monitoring under the stimulus of sunlight can identify the interfaces under the geomembrane with Newton’s cooling law, and the profiles of substances can be identified with valid thermal imaging sequences.

**Figure 10:**
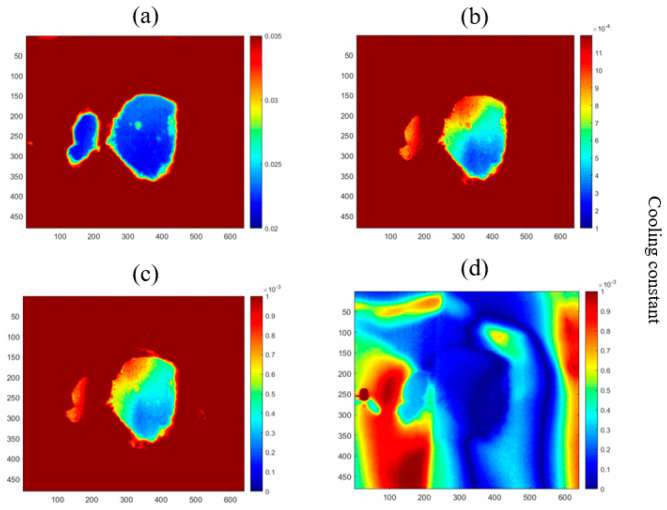
Comparison of cooling constant maps generated from different lengths of periods. (**a**) The sum of period 2 thermal image sequences. (**b**) The sum of 60 min thermal image sequences (6 frames). (**c**) The sum of 40 min thermal image sequences (4 frames). (**d**) A single frame of cooling constant maps in 40 min.

As shown in Figure 10, cooling constant maps were generated from different lengths of thermal image sequences in period 3 of Figure 8. Figure 10a is a cooling constant map that is generated from period 2 (07:07:55–17:11:15) in Figure 8. It presents a clear profile of the interfaces under the geomembrane. Figure 10b is generated from the first 60 min in period 2 of Figure 8. Six sets of cooling constant values are summed up to compare the contrast, where the small soil region is blurred in the map and the contrast between the soil region and non-soil region is not uniform, but the soil region still can be identified. In Figure 10c, the cooling constant map which is generated from the first 40 min of monitoring shows the profile of the soil region with a non-significant contrast. However, the soil regions still can be identified. For Figure 10d, which is generated from 10-min monitoring (two frames of thermal images), the whole image becomes blurred, and the profile of the soil region cannot be identified. These four figures reveal that a short thermal image sequence (around 40–60 min with 4–6 thermal images) can still demonstrate a reasonable profile of interfaces under the cover, even though some soil regions are blurred, and the cooling constant contrast is insignificant.

This study demonstrated that cooling constant maps from transient events in a day can be used to present subsurface substances. Figure 10d shows that summing the cooling constant values from multiple periods can reveal clearer soil profiles beneath the covers due to the accumulated cooling constant value difference between the soil and surrounding areas. For long-term monitoring, the number of transient temperature decay events increases over time, enabling better characterization of features beneath the covers and monitoring of scum accumulation. In addition, monitoring time or the selection of thermal image frames does not affect the continuous monitoring of covers. It eliminates the need to search for suitable frames and identify features from a single thermal image frame.

### 3.3. Classification of Different Interfaces Under the HDPE Geomembrane

**Figure 11:**
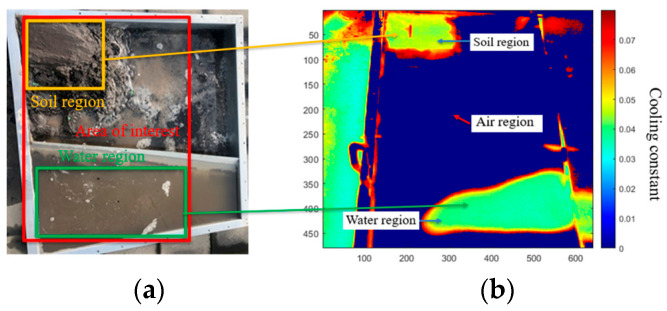
(**a**). Test rig filled with soil, air, and water at different regions under the HDPE geomembrane cover. (**b**). Cooling constant maps with three regions (soil region, water region, and air region) on the HDPE geomembrane.

Based on the above findings with the developed thermal imaging technique, an experiment was set up to identify three interfaces (air, water, and soil) under the geomembrane, which simulate the interfaces (biogas, sewage water, and scum) under the floating covers. As shown in Figure 11a, the three kinds of substances were filled in the test rig, where the water was isolated at the bottom by a barrier, and the soil block was built at the top left corner of the test rig. The whole set-up was exposed to sunlight during the daytime, and the monitoring lasted 40 min. A thermal image sequence that included four frames of thermal images was analyzed with (5).

When several interfaces exist at the undersurface of the geomembrane at the same time, it is difficult to distinguish interfaces based on the contrast in a contoured cooling constant map. Therefore, several unsupervised clustering-based machine learning image segmentation algorithms, such as K-means clustering, DBSCAN clustering, and GMM clustering were deployed to segment the cooling constant map into several parts based on the values of the cooling constant. As shown in Figure 11b, the cooling constant map was plotted after the calculation with Newton’s cooling law. Due to the uneven test specimen surface profile, only part of the geomembrane contacted the water. In this figure, the areas of each region are demonstrated on the map. In addition, the air region can be distinguished from another two regions with a large contrast. However, the soil and water regions cannot be distinguished due to the small difference in the cooling constant, and the presence of each region in the map depends on the threshold of the cooling map.

**Figure 12:**
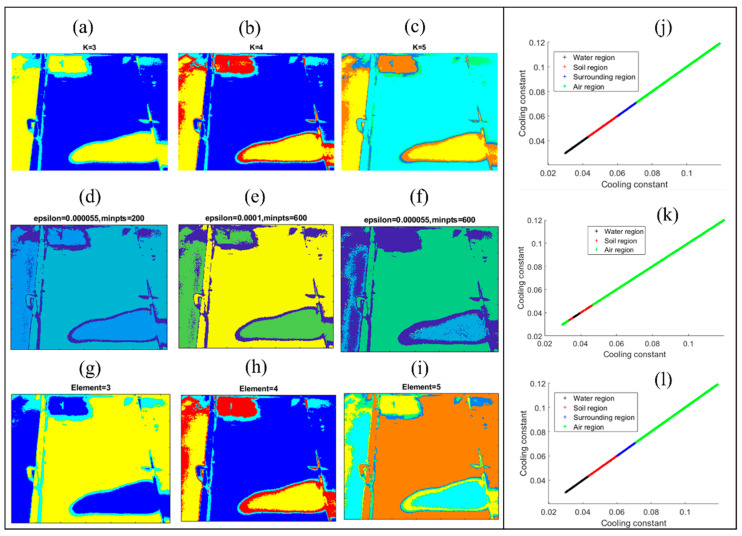
(**a**–**c**) Profiles of each region after K-means clustering segmentation. (**d**–**f**) Profiles of each region after DBSCAN clustering segmentation. (**g**–**i**) Profiles of each region after GMM clustering segmentation. Classification of regions using different clustering algorithms. (**j**–**l**) Cooling constant data distribution of each cluster in K-means, DBSCAN and GMM methods.

Figure 12 shows the image segmentation results with different clustering algorithms. For each clustering algorithm, the cluster specifications were altered for comparison. The K-means clustering method was first deployed for analysis, and the results are shown in Figure 12a–c. As discussed in Section 2.2, the number of clusters is set as 3, 4, and 5 to compare the segment results. Figure 12a shows the result with K = 3, where the air region is distinguished from another two regions, but the water and air regions are classified as the same region. Figure 12b shows the result with K = 4, where all three regions are recognized, and the fourth region is the surrounding region. When K = 5, Figure 12c shows a similar result with K = 4, while contours around each region are divided into more layers. This is because the lateral thermal conduction results in a varied cooling constant at the edge of each region. Compared to the cooling constant map in Figure 11, figures with K = 4 and 5 present agreed profiles of each region. As in the experiment, there are three interfaces under the geomembrane and a surrounding environment, and the data in the cooling constant map were categorized into four groups (K = 4).

The classification performance for three clustering algorithms is summarized in Table 2. Figure 12j shows the cooling constant data distribution ranges for each cluster. It can be seen that the water region has the lowest cooling constant values, and the soil region has a higher cooling constant than the soil region. The air region has the highest cooling constant values and is the largest region in the image. The surrounding region is located at the edge of each region and some surrounding parts are in the image. Hence, this algorithm successfully predicted different interfaces under the HDPE geomembrane.

The result of the DBSCAN clustering method is shown in Figure 12d–f. Figure 12d shows the processed image with = 0.000055 and minpts = 200. The soil region, the water region, and the air region can be distinguished in the image. As the DBSCAN method automatically determines the number of clusters, the image is divided into three clusters and the surrounding region is not recognized. Figure 12e shows the result when =0.0001 and minpts = 600. The data are divided into three clusters again and the air region is distinguished, while the air and water regions cannot be distinguished. Figure 12f shows a similar result to Figure 12d with = 0.000055 and minpts = 600. The detailed data distribution of each clustered region with the DBSCAN clustering algorithm are shown in Figure 12k, where the water region data take the smallest area in the image, and the data are mainly distributed at the water region and some of the surrounding regions. Soil region data are distributed at the soil region, the outer layers of the water region, and some parts of the surrounding region. It was found that some of the soil region data are smaller than the water region data because some of the data in the water region were classified as soil region data. Similarly, some of the data in the water region are classified as the air region data, resulting in some of the low cooling constant values data being classified as the air region data. Even though some of the points were not classified correctly, the DBSCAN method can segment the cooling constant map and present profiles of each region.

The segmentation result with the GMM method is shown in Figure 12g–i. Figure 12g shows the result with three components, and the air region can be distinguished from the other two regions (water region and soil region). Soil water regions are not distinguished since they are classified as the same components and some other areas are classified as the third component. Figure 12h shows the result with four components, presenting a suitable classification with all three classified regions. Figure 12i can also classify all three regions, and the edges of the soil region and the water region are further classified into more layers. Similar to the K-means cluster methods, the GMM method with four components presents a suitable segmentation result, and the data distribution of each cluster is shown in Figure 12l, where the soil region data take up a larger area than the water region data because the out layer of the water region is classified as the soil region. The result shows that the GMM method can be used to segment the cooling constant map according to the cooling constant values. Table 2 shows the comparison between the clustering-based thermography method and several other classic thermography methods. The proposed clustering-based thermography method offers significant advantages over traditional techniques such as the Peak Temperature Contrast Method, the Logarithmic Peak Second Derivative Method, and the Least-Squares Fitting Method. The proposed clustering-based method can adaptively group pixels or regions based on thermal profile similarities, making it robust against noise and effective for analyzing complex thermal patterns. By leveraging unsupervised learning, this method avoids the need for predefined models or assumptions, allowing it to handle diverse materials and defect types. Furthermore, its ability to identify subtle and heterogeneous defects in thermographic data positions it as a versatile and efficient approach for defect detection, especially in scenarios with varying thermal behaviors or limited prior knowledge about the inspected structure.

So far, it can be concluded that the proposed clustering-based thermal imaging technique can be used to identify interfaces under the HDPE geomembrane, and the monitoring result can be obtained from a relatively short period of monitoring the stimulus of solar radiation.

The proposed thermographic method, which relies on the thermal contact of substrates with the underside of the HDPE geomembrane, precludes the occurrence of overlapping substrate profiles due to the distinct thermal signatures they produce. This distinction is crucial as it allows for the segregation of different scum states, such as fluffy and solidified, within clustering analysis. While these variations present a challenge, they are not considered overlapping but rather different states of the same substance, which the proposed method can differentiate.

## 4. Conclusions

This study addresses the critical challenge of monitoring waste substrate digestion under HDPE geomembranes in sewage treatment plants, which is essential for optimizing biogas collection and maintaining geomembrane integrity. This research presents a non-intrusive monitoring approach, offering a significant advancement in the operational and environmental management of these large-scale structures. A clustering-based thermography technique has been experimentally investigated for detecting multiple substances under the HDPE geomembrane material. The proposed thermal imaging uses the daily transient temperature responses from natural solar radiation to predict the profiles of substances beneath covers. The key findings of this study are summarized as follows:

- Outdoor thermography, based on the transient temperature changes from solar radiation, efficiently identifies multiple attachment profiles under the HDPE geomembrane.
- The proposed thermography technique can identify various states of matter including solid, fluid, and gas under the geomembrane using the K-means clustering algorithm, the DBSCAN clustering algorithm, and the GMM clustering algorithm, respectively. The interfaces are classified based on cooling constant values and their locations and areas are presented on a map through machine learning image segmentation.

The limitation of this work comes from a lack of quantitative verification of areas on the cover. The contact area between substances and covers cannot be measured because the contact area immediately changes if the cover is lifted. To address these limitations, remote sensing technologies such as ground-penetrating radar (GPR) or electromagnetic induction (EMI) can be employed to assess subsurface contact areas without disturbing the geomembrane. Furthermore, advanced computational modeling can simulate the interactions between the substances and the covers, providing predictive insights into the contact area changes.

This quasi-active thermography is beneficial for wastewater treatment plants, allowing the recognition of substances through the opaque covers without lifting the covers. Additionally, thermography combined with ambient weather information offers valuable insights for large-scale structures’ structural health monitoring.

## Figures and Tables

**Table 1:** Manufacturer data of experiment instruments.

FLIR A615 IR Camera	Specifications
IR resolution	640 × 480 pixels
Field of view (FOV)	25° × 19°
Full image frequency	50 Hz
Noise equivalent temperature difference (NETD)	<0.05 °C @ +30 °C (+86 °F)/50 mK
Sampling rate	10 min/read
Detector type	Focal plane array (uncooled microbolometer)
Measurement accuracy	±2 °C
Emissivity correction	0.01–1
Object temperature range	−40–150 °C
**Fluke 287 multi-meter thermal probe**	**Specifications**
Operating temperature	−20 °C–55 °C
Mass	871 g
temperature resolution	0.1 °C
Accuracy	±1%
Sampling rate	30 min/read
**Apogee SP-110 pyranometer**	**Specifications**
Sensitivity	0.2 mV/Wm^−2^
Output range	0–400 mV
Mass	90 g
Sampling rate	10 min/read

**Table 2:** Comparison of thermography signal processing methods.

Methodology	Computational Complexity	Sensitivity to Noise	Accuracy for Defect Detection
Our method	Low	Moderate	Moderate
Peak Temperature Contrast Method [55]	Low	Moderate	Easy
Logarithmic Peak Second Derivative Method [56]	Moderate	High	Moderate
Least-Squares Fitting Method [57]	High	Low to Moderate	Complex

## Data Availability

The data will be available upon request.

## Abbreviations

HDPE: High-density polyethylene; MWC: Melbourne Water Corporation; CAL: Covered anaerobic lagoons; UAV: Unmanned aerial vehicle; DEMs: Digital elevation models; TSR: Thermography signal reconstruction; LPSD: Logarithmic Peak Second order Derivative; DBSCAN: Density-based spatial; GMM: Gaussian mixture model; WCSS: Within-cluster sum of squares; PV: Photovoltaic.
